# Conditioned up and down modulations of short latency gamma band oscillations in visual cortex during fear learning in humans

**DOI:** 10.1038/s41598-022-06596-8

**Published:** 2022-02-16

**Authors:** Alejandro Santos-Mayo, Javier de Echegaray, Stephan Moratti

**Affiliations:** 1 Department of Experimental Psychology, Psychology Faculty, Universidad Complutense de Madrid, 28223 Pozuelo de Alarcón, Madrid Spain; 2 Laboratory of Cognitive Neuroscience, Center for Biomedical Technology, Universidad Politécnica de Madrid, Madrid, Spain; 3 Laboratory of Clinical Neuroscience, Center for Biomedical Technology, Universidad Politécnica de Madrid, Madrid, Spain

**Keywords:** Cognitive neuroscience, Emotion, Learning and memory, Sensory processing, Visual system

## Abstract

Over the course of evolution, the human brain has been shaped to prioritize cues that signal potential danger. Thereby, the brain does not only favor species-specific prepared stimulus sets such as snakes or spiders but can learn associations between new cues and aversive outcomes. One important mechanism to achieve this is associated with learning induced plasticity changes in sensory cortex that optimizes the representation of motivationally relevant sensory stimuli. Animal studies have shown that the modulation of gamma band oscillations predicts plasticity changes in sensory cortices by shifting neurons’ responses to fear relevant features as acquired by Pavlovian fear conditioning. Here, we report conditioned gamma band modulations in humans during fear conditioning of orthogonally oriented sine gratings representing fear relevant and irrelevant conditioned cues. Thereby, pairing of a sine grating with an aversive loud noise not only increased short latency (during the first 180 ms) evoked visual gamma band responses, but was also accompanied by strong gamma power reductions for the fear irrelevant control grating. The current findings will be discussed in the light of recent neurobiological models of plasticity changes in sensory cortices and classic learning models such as the Rescorla–Wagner framework.

## Introduction

Discriminating potentially harmful stimuli from background noise, such as detecting a snake hidden in the grass, is crucial for survival. During the course of evolution species-specific stimulus categories have been evolved, that are innately perceptually prioritized. For example, in humans stimulus categories such as insects (e.g. spiders), reptiles (e.g. snakes) and highly social relevant stimuli (e.g. fearful faces) represent such to-be-prepared cues^1–3^. However, in ever-changing environments organisms also must be capable to learn to perceptually prioritize new stimuli that represent or alert danger and do not form part of the evolutionary prepared stimulus sets. For example, previously innocuous stimuli that have been paired with aversive events (e.g. a loud noise burst or a mild electric shock) can acquire the same motivational relevance as natural motivators; a process called Pavlovian fear conditioning. Importantly, stimuli that acquired fear relevance by learning are also perceptually favored as these stimuli also evoke autonomous reactions associated with orienting responses and modulate neural gain in early sensory cortices^4–6^.

Inspired by animal models in the auditory domain, recently there has been a great interest in how short-term plasticity changes in the human visual cortex alter the sensory representation of visual conditioned stimuli during associative learning^7–9^. From a behaviorally adaptive point of view, enhanced neural gain in sensory systems facilitates a better detection of acquired fear relevant stimuli among other potential distractors. However, best discrimination between fear relevant and irrelevant stimuli would be achieved not only by facilitating the neural processing of fear associated stimuli, but also by decreasing neural activity of fear irrelevant stimuli. For example, in humans it has been shown that orientation sensitive visual cortex representations of Gabor patches that are similarly orientated as a fear relevant Gabor patch are suppressed possibly by the retuning of orientation selectivity of visual cortex neurons^10^. Further, a recent MEG study has shown that during fear conditioning neural gain increases for a fear relevant visual stimulus was accompanied by a diminution of neural activity for the fear irrelevant cue^5^. These studied used steady state visual evoked potentials (ssVEPs, EEG) or fields (ssVEFs, MEG) that represent evoked neural oscillations driven by a visual flicker at a certain frequency. Steady state responses have the advantage of high signal to noise ratios but lack sufficient time resolution in order to determine if these opposing neural gain modulations occur at short latency visual cortex responses. Additionally, these studies showed rather late latency effects on steady state power changes evoked by flickering stimuli just before US onset^6^. Further, ssVEPs or ssVEFs likely represent reentrant oscillatory activity from higher to lower order visual cortex and it is difficult to disentangle at which processing stage these neural gain modulations occur^11^.

**Figure 1:**
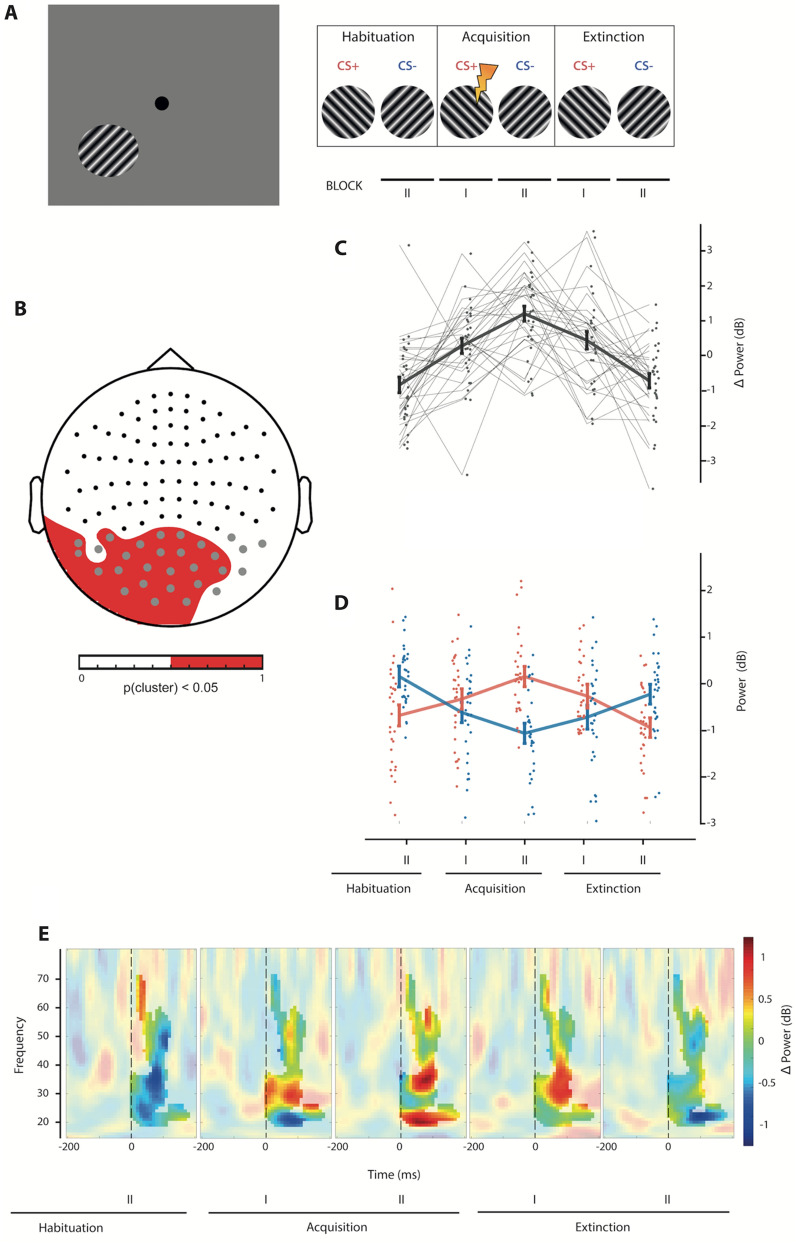
Sensor space analysis of evoked gamma band power. (**A**) The left panel represents the lower left visual field presentation of a sine grating. The right panel depicts the three phases of the experiment (habituation, acquisition, and extinction). Each phase was divided into two blocks. (**B**) The topography of the significant quadratic contrast cluster of the CS+/CS− differences across experimental phases is shown. The colorbar indicates in red which sensors pertain to the cluster (*p*_cluster_ < 0.05). (**C**) Mean power differences (CS+ minus CS−) across significant sensor, time, and frequency triplet clusters are shown (grey line). The error bars represent standard errors. Further, a paired observation plot for each subject (connected dots, N = 30) was overlayed to the contrast plot. (**D**) Mean power changes (dB) to the pre-stimulus baseline across the same sensor, time, and frequency triplet clusters as in C are shown for the CS+ (red) and CS− (blue) conditions separately and for each block. The dots represent individual participants (N = 30). The error bars represent standard errors. (**E**) Mean spectral power differences (CS+ minus CS−) in the time–frequency domain across significant cluster sensors for each experimental phase are shown. The highlighted areas represent the time–frequency dimensions of the significant cluster. The colorbar represents power changes in dB.

Here, we extend these previous findings by investigating if such opposing adaptive neural gain changes for fear relevant and irrelevant visual stimuli, that facilitate best stimulus discrimination, occur during very early processing stages in the visual cortex. Thereby, short-latency neuromagnetic evoked gamma band oscillations usually observed during visual processing^12,13^ were recorded with MEG during a delayed discriminative Pavlovian fear conditioning procedure. Thereby, two orthogonally oriented visual sine gratings served as the conditioned (CS+) and the control stimulus (CS−). After a habituation phase in that no CS had been paired with an aversive loud acoustic noise (unconditioned stimulus; US), only the CS+ was paired with the US during CS-US association learning in an acquisition phase. Finally, during extinction none of the conditioned stimuli were paired with the US (see Fig. 1A and “Methods” for a detailed description).

Neuromagnetic evoked gamma band responses to such sine patches represent early stages of visual information processing in humans^14,15^. Further, it has been shown that increased gamma band oscillations in sensory cortices can modulate sharpened representation of the CS+ by short-term plasticity changes such as shifts in frequency tuning and changes in receptive field plasticity of auditory cortex neurons during fear conditioning^16–18^. Similarly, gamma power increases have been observed during changes in orientation selectivity of visual cortex neurons^7,19,20^. Therefore, neuromagnetic gamma band modulations as recorded by MEG in humans may represent a good proxy for processes that are related to these short-term plasticity changes. Neural mass activity in the gamma frequency range, that can be recorded with MEG, may indicate shifts of orientation tuning in visual cortex towards the CS+ and away from the CS− patch orientation. Thus, gamma power increases and decreases, may reflect such an orientation re-tuning process. As efficient discrimination between fear relevant (CS+) and irrelevant information (CS−) should occur fast, we hypothesize that such opposing gamma power modulations emerge quickly after stimulus onset.

Further, cortical source estimation of the corresponding neuromagnetic signal changes should localize to the lower tier of the visual cortex. Critically, reduced gamma band responses for the fear irrelevant stimulus should increase to baseline levels during fear extinction learning in order not to be attributable to habituation processes as a result of repeated stimulus presentation. Taken together, short latency opposing gamma power modulations at low levels of the visual system could be associated with short term plasticity processes facilitating fast adaptive visual discrimination of acquired motivational information in humans.

## Results

### Unawareness of conditioning and levels of vigilance

In order to maintain a certain level of the participants’ attention and to prevent developing awareness of the CS-US contingency (the aware detection of the rule that the CS+ predicts the US), volunteers had to respond to random color changes of a centered dot (black to grey) after CS presentation. The total detection rate confirmed a high state of vigilance during the experiment (total: 95.56% ± 8.32 s. e. m.). Detection rates did not depend on the experimental phase (habituation, acquisition, extinction, here blocks I and II were collapsed for this analysis) or condition (CS+ vs. CS−) as indicated by a non-significant interaction experimental phase by condition (F(2, 58) = 0.25, *p* = 0.771, ε = 0.97, η^2^ = 0.009). However, with respect to reaction times (detection speed) an interaction of experimental phase (habituation, acquisition, and extinction) by condition (CS+ vs. CS−) was observed (F(4, 58) = 3.44, *p* = 0.052, ε = 0.77, η^2^ = 0.106). This interaction was explained by faster reaction times in CS+ (555.68 ms ± 28.26 s. e. m.) than in CS− trials (588.10 ms ± 21.81 s. e. m.) during acquisition (t(29) = − 2.43, *p* = 0.022, d = − 0.44). During habituation (t(29) = 0.37, *p* = 0.717, d = 0.07) and extinction (t(29) = 1.21, *p* = 0.237, d = 0.22) reaction times did not differ after CS+ and CS− presentations.

The CS-US contingency was not detected by any of the participants as by the end of the experiment no one (N = 30) could verbally express the awareness of any rule that predicted the administration of the US. Therefore, none of the volunteers reached the second stage of the questionnaire where the CS+ had to be identified.

### Evoked neuromagnetic gamma band responses

We hypothesized that best discriminative representations of the fear relevant CS+ and fear irrelevant CS− in visual cortex will be associated with short-latency evoked oscillatory gamma band increases for the CS+ and decreases for the CS− during the learning phase of the experiment compared to baseline conditions (habituation and extinction). Therefore, a time–frequency analysis was conducted at occipital and parietal sensors, usually showing an early gamma band response during visual processing (see “Methods” section). Further, a facilitation of visual processing during acquisition of the CS-US association for the CS+ and a decrease of CS− related activity should be best represented by an increase of gamma band power differences between the CS+ and CS− from habituation to acquisition phases followed by a decrease of power differences for extinction trials. Therefore, a quadratic contrast (see “Methods” for details) across experimental phases was fitted to the CS+ and CS− power differences, thus testing the interaction between quadratic CS+ and CS− gamma power changes and experimental phase. The significance of this contrast was evaluated using a non-parametric permutation procedure (see “Methods”).

A sensor cluster at medial occipital sensors indicated a statistically significant quadratic contrast across experimental phases for the CS+ and CS− differences (cluster-based permutations, summed F = 6536, *p* < 0.05; best fit at gradiometers MEG 2142 + 2143, frequency 52.2 Hz, time 0.1 s, F(1, 29) = 17.8, *p* < 0.001, η^2^ = 0.381). The topography of the resulting cluster is shown in Fig. 1B. The quadratic effect spanned from stimulus onset to 180 ms and ranged between 18.9 Hz and 71 Hz. The CS+/CS− difference was biggest during the second block of acquisition (see Fig. 1C) and returned to habituation levels during extinction. The paired observation plots for each individual subject in Fig. 1C indicated a robust quadratic pattern of CS+ and CS− gamma power differences across experimental phases.

Critically, the quadratic modulation of CS+ and CS− differences across experimental phases was not only driven by a progressive increase of gamma band responses during acquisition for the CS+ (see red line in Fig. 1D), but also by CS− gamma band power decreases (see blue line in Fig. 1D). Accordingly, CS+ mean gamma power across significant sensor by frequency by time clusters (see above) followed a quadratic response profile across experimental conditions (quadratic fit: F(1, 29) = 13.89, *p* < 0.001, η^2^ = 0.324). In contrast, the CS− condition was characterized by a progressive decrease of gamma responses during acquisition and a return to baseline during extinction (quadratic fit: F(1, 29) = 44.46, *p* < 0.001, η^2^ = 0.605). Finally, Fig. 1E depicts the five time–frequency power differences between the CS+ and CS− across significant sensors for each experimental phase (habituation block II, acquisition block I and II, and extinction block I and II).

### Cortical source localization of gamma response interaction

**Figure 2:**
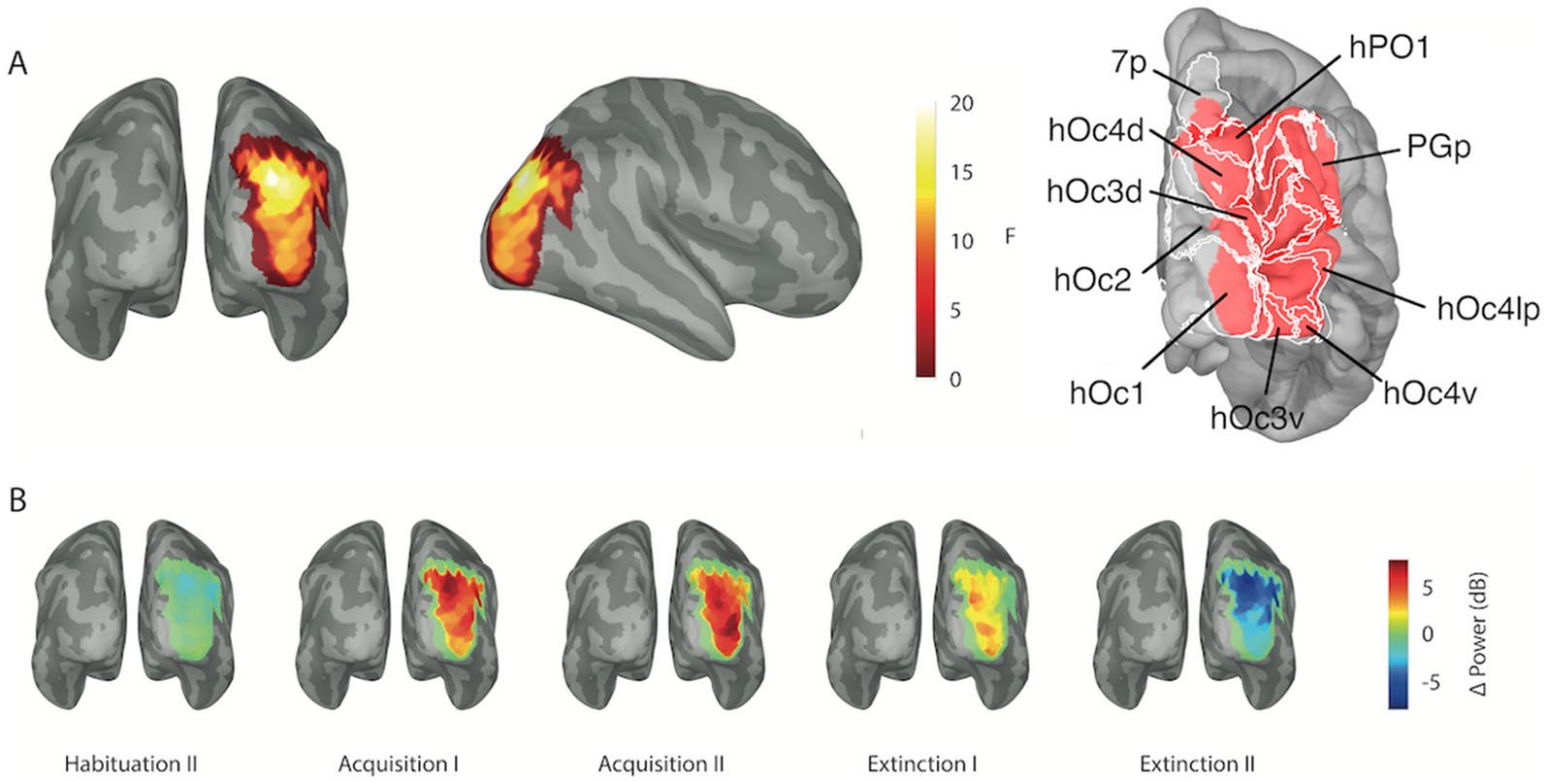
Source space analysis of evoked gamma power. (**A**) The significant cluster of the quadratic fit for the CS+/CS− differences across experimental blocks is shown (back and right lateral view). The colorbar indicates the F value of the quadratic contrast for the CS +  and CS− power differences across experimental phases. The brain surface is inflated to visualize better all F values. On the right the significant source cluster is shown in red overlayed with cytoarchitectonically identified human visual areas (white shaded areas) derived from the MNI aligned Jülich brain atlas. The right hemisphere from a back view is shown. Here, the brain surface is not inflated to better visualize the anatomy. (**B**) Gamma power response differences between the CS +  and the CS− for sources within the aforementioned significant cluster across experimental blocks are depicted (inflated brain surface). The colorbar represents gamma power CS + /CS− differences in dB.

Although the hypothesized effects were observed at posterior MEG sensors, a linearly constrained minimum variance (LCMV) beamformer was applied (using all gradiometers) in order to localize the quadratic gamma power difference (CS+ vs. CS−) modulation in cortical source space. The time resolved LCMV beamformer source waveforms between stimulus onset and 180 ms after stimulus presentation were submitted to an FFT and mean power between 18.9 and 71 Hz was extracted at cortical source locations (baseline corrected to a corresponding time window in the pre-stimulus interval, see “Methods”). The same quadratic contrast and permutation procedure as for the sensor level analysis was fitted to the CS+ and CS− power differences across experimental conditions. A significant source cluster revealed enhanced gamma power differences (CS+ minus CS−) during acquisition from areas human occipital 1 (hOc1, V1), hOc2 (V2), hOc3v (ventral), hOc3d (dorsal), hOc4d, hOc4v, hOc4lp up to occipito-parietal visual areas (hPO1, PGp, 7P) in the right hemisphere (cluster-based permutation, summed F = 747; *p* < 0.05, maximum F value: F(1, 29) = 21.891, *p* < 0.001, η^2^ = 0.43; Fig. 2A, B). The LCMV beamformer showed that the observed sensor level effects were attributable to lower and middle-tier visual areas.

## Discussion

Here, we show that conditioned neural gain increases and decreases in the human visual cortex were greatest around 100 ms after stimulus onset for acquired fear relevant and irrelevant stimuli, respectively. This was reflected by corresponding evoked gamma band power modulations between 18.9 and 71 Hz from lower to mainly middle tiers of the visual cortex. Thereby, oscillatory neuromagnetic gamma band activity not only increased for the CS+ during the acquisition phase of the CS-US association, but also decreased for the CS−. Critically, both, CS+ and CS− related evoked gamma power returned to pre-conditioning levels during extinction. Our findings not only replicate previous observations of short-term plasticity changes in the visual cortex by learning, but significantly extend these reports by demonstrating that these changes occur very fast within the first 180 ms peaking at around 100 ms after stimulus onset at lower to middle-tier areas of the visual cortex including the lateral part of V1. In contrast, we did not find any gamma power modulations for induced gamma band responses (see supplementary Fig. S1). This is in line with the notion that induced late latency gamma power changes have been related to cognition and higher order perceptional processes^14,15^, whereas short-latency evoked gamma band oscillation represent the processing of basic stimulus features^15,21^. Further, the evoked gamma power modulations were specific to the gamma band. There were no differences in the lower frequency bands, nor could the gamma activity changes be explained by the evoked C1 component (see supplementary Fig. S2).

However, Fig. 1D suggests that for some reasons evoked mean gamma power across the significant sensor clusters was higher for the CS− during the habituation phase (t(29) = 3.80, *p* < 0.001, d = 0.71, see Fig. 1D). As both CSs were presented randomly in the left and right visual hemifields and the grating orientation was counterbalanced across participants, this difference (although associated with a big effect size) may be due to random activity fluctuations. However, this difference does not affect the interpretation of our results as the main interest here is the modulation of power differences between the CS+ and CS− across the experimental phases. Critically, evoked gamma power was greater for the CS+ than for the CS− during the second acquisition phase (t(29) = 5.50, *p* < 0.001, d = 1.02) despite the gamma power differences during habituation (see supplementary Fig. S3).

Previous studies that showed opposing neural gain modulations in the visual cortex by learning could not directly assess short-latency visual processing, because these studies utilized steady state responses that reflect visual cortex activity across several seconds before US appearance^5,6,10^. However, Keil et al.^21^ also reported gamma band increases between 60 and 90 ms after CS+ onset during fear conditioning of visual gratings paralleling our results. In line with our study, Keil and collaborators^21^ also observed the biggest learning induced gamma band modulations during the second acquisition block indicating increased neural gain for the fear relevant stimulus as a result of ongoing learning. However, these authors did not report gamma power reductions for the CS− during acquisition. This might be due to the utilization of unpleasant pictures as US. Here, we used a 90 dB SPL noise that may be more aversive and therefore probably induced more differential effects with respect to opposing neural gain modulations of the CS+ and CS− in the visual cortex.

Conditioned gamma band response reductions for the fear irrelevant CS− as observed here accords with animal studies in the auditory domain that showed similar opposing activity changes for the CS+ and CS− in the amygdala, thalamus and auditory cortex^22–24^. At the cortical level these processes have been associated with frequency tuning shifts of neurons in the auditory system. Further, Headley and Weinberger^17^ have shown that neurons that code the tone frequency of the CS+ exert gamma frequency boosts associated with increased phase-locking of single spike trains to gamma cycles. In contrast, neurons that code frequencies far from the CS+ showed decreased phase-locking of spike occurrence. In line with our observation of increased CS+ gamma power and diminished CS− gamma band activity the opposing phase-locking patterns reported by Headley and Weinberger^17^ may represent a mechanism of sharpening representations and down-stream processing of behaviorally relevant stimuli in comparison to irrelevant ones. Importantly, increased CS+ gamma band oscillations after learning predicted the strength of associative memory and receptive field plasticity underscoring the behavioral relevance of these processes^16^.

Critically, Galuske, Munk, and Singer^7^ observed a similar mechanism in the cat’s visual cortex showing that cholinergic driven increased gamma band activity predicted changes of orientation preference in neural populations (see also^8^). The spatial extent of conditioned orientation domains was not only associated with increased gamma band responses to a conditioned orientation but also with a decrease of signal strength for the orthogonal orientation paralleling the opposing gamma power modulations in our study.

The conditioning effects here were located mainly in middle-tier visual up to occipito-parietal areas with the involvement of the lateral part of V1 (hOc1). This is in line with the observation that the C1 component, that reflects the first afferent to visual cortex, was not modulated by conditioning in our study (see supplementary material and Fig. S2). However, it is widely accepted that orientation selective neurons exist in visual areas beyond V1 and V2 (for example see^26,27^).

Interestingly, conditioned increments and decrements of gamma power changes in the visual cortex for the CS+ and CS−, respectively, cannot only be explained by neurobiological informed models, but also formalized by classic learning theories^25^. The early Rescorla–Wagner model of how associative strength develops during the CS-US association learning and results in excitatory responses for the CS+ also emphasizes the acquisition of conditioned response inhibitory properties of the CS−^28–30^. Thereby, the final associative strength λ between the CS+ and US must be ideally reach 1. This λ is approached during trial-by-trial learning that follows a decreasing linear function of the difference between the accumulative associative strength V and this predetermined λ value: ΔV = Φ(λ − V), whereby Φ stands for US intensity. In contrast, the final associative strength for the CS− must be ideally 0. However, as both, the CS− and the CS+, are always presented together with the background and experimental context, this context is also reinforced during CS+ presentations and gains some associative strength with respect to the US. Now, when the CS− is presented together with the context, the final accumulative associative strength V for the CS− must be negative in order to counteract the conditioned excitatory response context. This is how the CS− converts itself into a conditioned response inhibitor as the Rescorla–Wagner model assumes that association learning encompasses stimulus combinations^30^.

One issue arising during the review process of this work is, if the observed conditioning effects of early evoked gamma power modulations depend on spatial attention. However, it is unlikely that the observed effect were driven by spatial attention. First, participants were highly engaged in a central target detection task (detection rate 95.6%), whereby the CS+ and CS− were presented in the left and right lower visual hemifields. Critically, the detection rates were not modulated by experimental phase (habituation, acquisition, extinction) and condition (CS+, CS−) as no interaction between these factors was observed. In contrast, detection speed differences between the CS+ and CS− differed between experimental blocks. However, this interaction was weak and the participants detected the luminance changes of the central fixation cross slightly faster in CS+ trials. If the CS+ captured more spatial attention than the CS−, participants should have been slower in detecting the central fixation cross changes in CS+ trials. Second, after the experiment the participants could not identify any rule that predicted the administration of the US. Third, the C1 component (see supplementary material and Fig. S2) was not modulated by fear learning. It has been reported that the C1 amplitude can be modulated by spatial attention^31^. Finally, as alpha band power changes have been conceptualized to index attentional processes of inhibition and facilitation of stimulus processing^32^, we assessed if induced neural oscillations in the alpha frequency range were modulated differently for the CS+ and CS− across experimental phases. No such modulations in the alpha frequency range was observed (see supplementary material and Fig. S4). Therefore, it is unlikely that evoked CS+ increases or CS− decreases in the gamma frequency range were driven by spatial attention.

Taken together, both, classic learning theory and neurobiological models of plasticity changes in early sensory cortex can explain our observation of opposing gamma band power modulations in visual cortex during fear conditioning of visual stimuli. Thereby, increased macroscopic neuromagnetic gamma band responses for the fear relevant sine grating of a certain orientation may reflect shifts of preferred orientation selectivity of visual cortex neurons towards the fear relevant orientation^7^. This increased recruitment of neurons preferably firing for the fear conditioned orientation probably is accompanied by increased gamma power as measured at a macroscopic level. This process would also lead to a reduction of gamma power for the orthogonally oriented sine grating that was fear irrelevant. Importantly, this results in a sharpened sensorial representation of the CS+ as neural activity is not only increased for the fear relevant but also decreased for the fear irrelevant CS−. Critically, we show that conditioned increases and decreases of neural gain control for fear relevant and irrelevant stimuli, respectively, occurs very fast after stimulus onset in the human visual cortex.

## Methods

### Subjects

A sample of 30 volunteers (22 females, 25 right-handed, mean age 23.9 years, range 20–37 years) participated in the study. All participants had normal or corrected to normal vision and no auditory anomalies. The study had full ethical approval from the local ethics committee (Comisión deontológica/Ethics Committee, Facultad de Psicología, Universidad Complutense de Madrid) according to the Declaration of Helsinki and all participants gave written informed consent. We confirm that all methods were performed in accordance with the relevant guidelines and regulations following the Spanish and European law (Ley Orgánica 15/1999 de Protección de datos de Carácter Personal LOPD y Real Decreto 994/1999).

### Stimuli

The experimental design consisted of a delay fear conditioning paradigm following immediate extinction divided in three phases: habituation, conditioning and extinction. The conditioned stimuli (CS) stimuli were two orthogonally oriented (45° and 135°) circular sine gratings of maximum contrast and consisted of 7 cycles across a visual angle of 2.2°. In order to optimally activate the cortical generators of the primary visual cortex we followed the procedure as suggested by DiRusso et al^33^. Thereby, stimuli were placed along a polar arc that was equidistant (2.62°) from a central fixation point and located at a polar angle of 45° below the horizontal meridian in the left or right visual field. We have chosen to stimulate the upper bank of the left and right visual cortex as these areas are better covered by the MEG sensors.

The assignment of the two gratings (oriented at 45° or 135°) to the CS+ or CS− condition was counterbalanced across subjects. A white acoustic noise (90 dB SPL) with instantaneous onset was binaurally presented through an air tube system (Neuroscan, El Paso, TX, USA) as the unconditioned stimulus (US). A centered black dot, subtending a visual angle of 0.17°, was depicted during the whole experiment as the fixation point. During the experiment the dot color could sometimes changed to grey (40% of maximum white luminance). All visual stimuli were presented in a magnetically shielded room by a video projector (Panasonic PT-D7700E) via a mirror system.

### Procedure

After subjects were familiarized with the MEG chamber, a written informed consent was given and signed. Then, subjects were seated into the shielded room placing their head under the MEG helmet. Subjects were instructed to focus on the black dot in the center of the screen and to press a button with their right or left index finger (counter balanced across subjects) as soon as the black dot changed its color to grey.

The experiment consisted of a discriminative delayed fear conditioning procedure with a habituation, acquisition, and extinction phase. Before habituation subjects were informed that no loud noise would be delivered. During habituation 40 CS+ and 40 CS− stimuli were presented in the left and right visual field, respectively, resulting in 80 trials per condition (CS+ and CS−). Each CS was presented for 0.8 s and the inter-trial interval varied randomly between 4 and 5 s. During 30 randomly chosen trials the black dot color changed to grey 0.8 s after CS onset requiring the participant’s responses.

Before the acquisition phase participants were informed that from now on a white acoustic noise burst would be presented from time to time. The acquisition block was identical to the habituation procedure, except that the US was always paired with CS+. Thereby the US (duration 0.400 s) was presented after 0.4 s after CS+ onset and co-terminated together with the CS+. The CS− was never paired with the US during acquisition. After the acquisition phase the extinction block started immediately without any instructions. The extinction block was identical to the habituation phase. After the experiment the participants’ awareness of the CS-US contingency was assessed. The questionnaire consisted of two parts. First, the participants were asked if they could verbally express any rule behind the US administration. If so, participants entered the second part where they had to identify which of the two sine gratings predict the US occurrence.

### Behavioral data

Detection rates and speed for the luminance changes of the central fixation cross were submitted to repeated measures ANOVAs with within subject factors of experimental phase (habituation, acquisition, and extinction collapsed across blocks I and II) and condition (CS+, CS−). Greenhouse–Geisser corrections were applied where appropriate.

### MEG data acquisition and pre-processing

MEG data was continuously recorded (600 Hz sample rate, 0.1–200 Hz online-filter) using a 306-channel (102 magnetometers, 204 orthogonal gradiometers pairs) system (Elekta-Neuromag^®^ VectorView, Helsinki, Finland, 2005). However, only gradiometer pairs were used for the final analysis as gradiometers are less noisy and we were not interested in deep sources. For artifact monitoring the electrooculogram (EOG) was recorded with electrodes attached above, below, left to and right to the outer canthus. The electrocardiogram (ECG) was recorded with electrodes placed at the left mid clavicle and lower right rib bone. Additionally, one electrode was attached to the left earlobe serving as the ground electrode. EOG and ECG were recorded simultaneously using standard Au electrodes (NSC Electromedicina) with the same sample frequency and on-line filters as the MEG data.

MEG data were spatially filtered using the temporal signal space separation (tSSS) algorithm implemented in Neuromag^®^ MEG software^34^. Eye blinks and cardiac artefacts were removed from data by independent component analysis (ICA-JADE) implemented in Brainstorm software^35^ (https://neuroimage.usc.edu/brainstorm). Noisy trials due to movement artifacts and horizontal eye movements as monitored by the EOG were determined by visual inspection and excluded from analysis. As we performed time–frequency analysis, the MEG data was not further off-line filtered. Then, peri-stimulus epochs of 1.8 s (1 s baseline and 0.8 s post-stimulus) were extracted for each condition (CS+ and CS−), experimental phase (habituation, acquisition, and extinction) and each subject. Left and right visual field conditions were collapsed. As learning effects were of principal interest in this study, experimental phases were further divided into first and second trial blocks (first 40, and second 40 trials). Finally, the second habituation, first and second acquisition and first and second extinction blocks entered into the analysis. The first trial of the acquisition and extinction conditions were removed from analysis.

### Spectral MEG analysis of evoked gamma band modulations

First, artefact free epochs for each condition (CS+ and CS−), experimental phase (habituation block II, acquisition blocks I and II, and extinction blocks I and II) and participants were averaged in order to obtain evoked MEG responses. Then, Morlet wavelets (7 cycles) were convolved with the averaged epochs at each gradiometer from 15 to 80 Hz in steps of 1 Hz in order to obtain evoked gamma band responses as we were only interested in early evoked gamma band modulations. Finally, evoked spectral perturbations within a 0.3 s post-stimulus interval were assessed by calculating power changes in dB with respect to 0.3 s baseline. The time–frequency analysis was performed using the fieldtrip toolbox^36^ (https://www.fieldtriptoolbox.org). Induced gamma band responses were also evaluated using the same method but applied to single epochs before averaging (see supplemental Material for details).

### Cortical source analysis

The source reconstruction of evoked MEG data epochs was based on a forward model based on an overlapping spheres head model^37^ using a canonical cortical mesh (3003 vertices) from the Montreal Neurological Institute^38^. Only gradiometer data was used for the cortical source reconstruction. The inverse solution was estimated with a linearly constrained minimum variance (LCMV) beamformer as implemented in the Brainstorm toolbox^35^. The data co-variance matrix for the common spatial filter across all conditions and experimental phases was calculated on the post-stimulus interval. Accordingly, a noise co-variance matrix was determined also across all conditions and experimental phases for the pre-stimulus interval. Then, the averaged MEG data epochs were convoluted with the post-stimulus spatial filter and z-normalized using the noise co-variance matrix in order to obtain a time domain Pseudo Neural Activity Index^35^ (PNAI) at three orthogonally oriented dipoles at each cortical vertex. Then, a Fast Fourier Transform (FFT) was applied to each PNAI time series of the three orthogonal dipoles at each vertex and the norm across the three orientations served as a power estimate at each cortical vertex. However, the FFT was restricted to the first 0.180 s after stimulus onset where significant gamma band modulations were observed at the sensor level (see “Results”). Then, an FFT in cortical source space was also calculated on a 0.180 s baseline interval (− 0.380 s to − 0.200 s) in order to estimate mean power changes in dB between 18.9 and 71 Hz (see “Results”) with respect to baseline.

### Statistical analysis

The aim of this study was to find an increase of CS+ and a decrease of CS− gamma power during learning of the CS-US association. Following the directed hypothesis of an increase of CS+/CS− differences from habituation over acquisition and a decrease during extinction a quadratic F contrast^39^ was fitted to the power changes (dB) at each time and frequency bin of the Morlet wavelet analysis at occipital sensors. Thereby, condition differences (CS+ minus CS–) for the five experimental phases (habituation II, acquisition I, acquisition II, extinction I and extinction II) were weighted by the contrast coefficients − 2.5, 1, 3, 1, and − 2.5 assuming biggest CS+ and CS− power differences during the second acquisition block.

A cluster-based permutation statistic was utilized in order to control for multiple comparisons. However, as early evoked gamma band responses are observed at posterior sensors, the analysis was restricted to occipital and parietal sensors (see Fig. 1B grey shaded sensors). Sensor clusters were formed when at least one adjacent time–frequency bin and spatial sensor neighbor indicated a significant quadratic F contrast fit (cluster alpha threshold of *p* = 0.05). Then, the F values were summed across the cluster. Thereafter, 10,000 random permutations between experimental phases for each MEG sensor under the Null hypothesis of no changes of CS+ and CS− power differences across the five experimental blocks were done. At each permutation step the same aforementioned cluster rule was applied and the maximum F cluster sum entered into a permutation distribution. Finally, empirical observed time–frequency sensor triplet F clusters with a sum exceeding the 97.5 percentile of the permutation distribution were considered as significant^40^. The permutation statistics was performed by using the fieldtrip toolbox. Exactly, the same procedure was applied to the induced gamma band responses at sensor space (see supplemental Material).

In cortical source space the same permutation statistics was used. However, as the data dimension had been reduced based on the time–frequency results at the sensor space level (mean power changes across 18.9 Hz and 71 Hz derived from an FFT of the first 0.18 s post-stimulus interval), only spatial F clusters of neighboring vertices of the canonical brain surface were considered during the permutation process. However, here a cluster alpha threshold of *p* = 0.01 was applied. In order to characterize the localization of the F cluster a cytoarchitectonic brain map (the Jülich brain^41^) was overlayed in MNI space using the Brainstorm toolbox^35^. Here, we report the brain areas defining the borders of the F cluster (see “Results” and Fig. 2).

## Supplementary Information

Supplementary Information.

## Data Availability

The data and analysis scripts can be obtained from the authors upon reasonable request.
