# Supplementary material for: Conditioned up and down modulations of short latency gamma band oscillations in visual cortex during fear learning in humans
Source: Sci Rep. 2022 Feb 16;12:2652. doi: 10.1038/s41598-022-06596-8 (PMC8850570; doi:10.1038/s41598-022-06596-8)
Supplement: Supplementary file 1 — Supplementary Information. [file 41598_2022_6596_MOESM1_ESM.docx]

**Conditioned up and down modulations of short latency gamma band oscillations in visual cortex during fear learning in humans**

Alejandro Santos-Mayo^1,2^, Javier de Echegaray^1,2^, and Stephan Moratti^1,2,3^

**Supplementary Information**

**Supplementary Methods**

Induced gamma band activity in sensor space was assessed exactly the same way as evoked gamma band power changes (see Methods in the main text), but instead of averaging the data epochs before applying the Morlet wavelet analysis, Morlet wavelets were convolved with each single trial for each condition, experimental phase and participants. Then, the single trial time frequency representations were averaged afterwards to obtain the induced gamma band power changes. Finally, the same cluster-based permutations statistics as with the evoked responses was applied (see Methods main text).

Further, we estimated the induced spectral perturbation in the alpha band range (8 Hz to 12 Hz) separately to evaluate possible attention effects. Thereby, the same approach as described above was utilized (single trial power analysis) but restricted to the alpha band. However, for the statistical analysis we averaged across the frequency bins from 8 Hz to 12 Hz, across the post-stimulus time interval (0 ms to 200 ms) and across the posterior sensor cluster as observed for the evoked gamma band modulations (see main text). Then the mean alpha power changes in dB were submitted to a repeated measures ANOVA with the within subject factors experimental phase (habituation block II, acquisition blocks I & II, and extinction blocks I & II) and condition (CS+, CS-). The Greenhouse-Geisser correction was applied were appropriate.

Further, we calculated the evoked oscillatory responses in the lower frequency bands (6 Hz to 40 Hz) in order to test if the evoked gamma band modulations were specific to this frequency range. The same statistical test as for the evoked gamma band modulations was applied. Finally, we also tested if the evoked responses in the time domain during this short-latency period (the magnetic counterpart of the C1 component) could explain the evoked gamma power differences. The C1 components were assessed using a bandpass (0.5 Hz to 40 Hz) and onl a highpass filter (0.5 Hz). The same cluster-based permutations statistics as in the previous assessments were utilized to estimate C1 amplitude modulations.

**Supplementary Results**

Figure 1S shows the results for the induced gamma band activity. Although a cluster emerged at posterior sensor positions, this cluster did not reach statistical significance (summed F = 6639, p = 0.177).


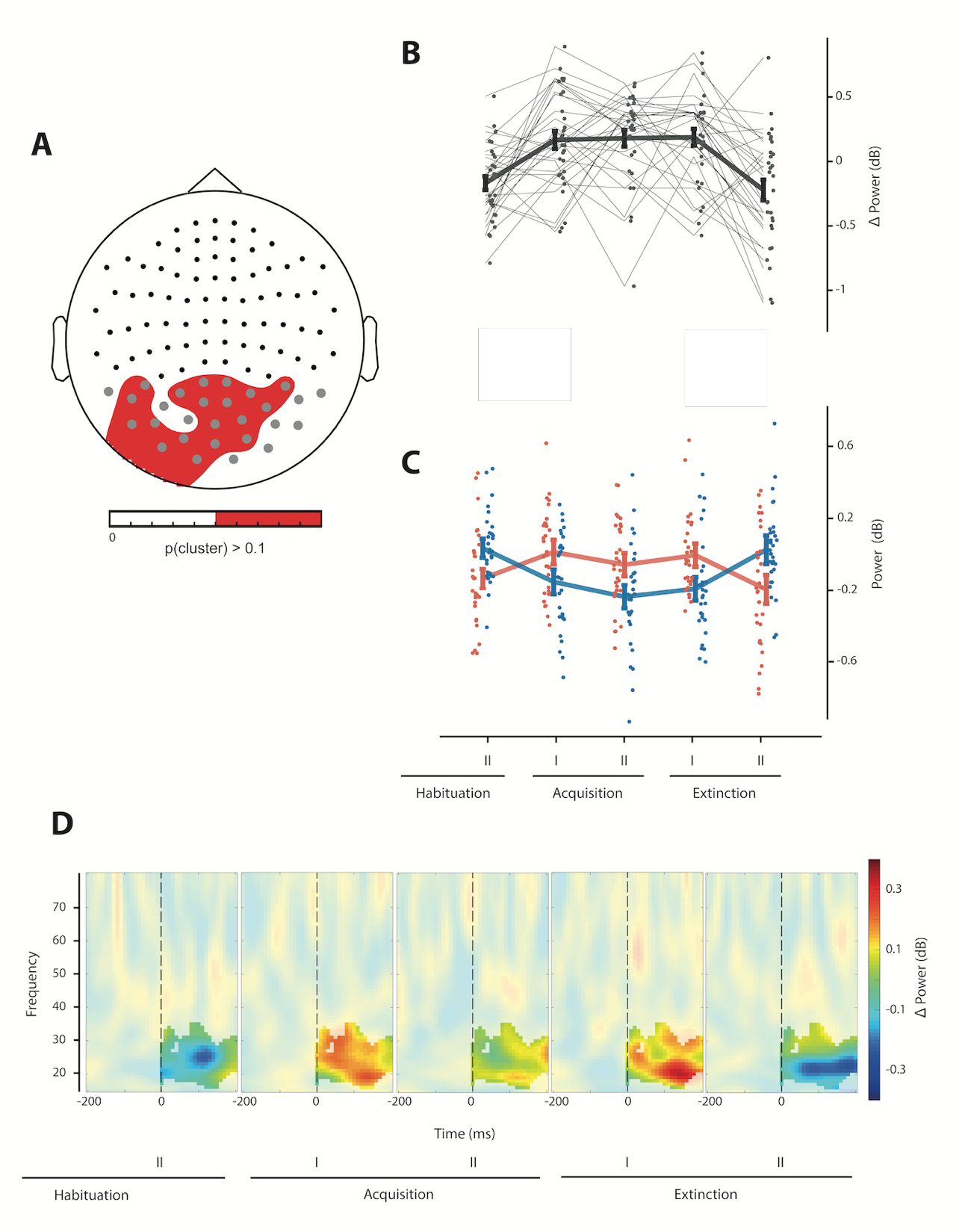


**Figure 1S: Sensor space analysis of induced gamma band power. (A)** The topography of the quadratic contrast cluster of the CS+/ CS- differences across experimental phases is shown. The colorbar indicates in red which sensors pertain to the cluster (p_cluster_ = 0.177). **(B)** Mean power differences (CS+ minus CS-) across the sensor, time, and frequency triplet clusters are shown (grey line). The error bars represent standard errors. Further, a paired observation plot for each subject (connected dots) was overlayed to the contrast plot. **(C)** Mean power changes (dB) to a pre-stimulus baseline across the same sensor, time, and frequency triplet clusters as in D are shown for the CS+ (red) and CS- (blue) conditions separately and for each block. The dots represent individual participants. The error bars represent standard errors. **(D)** Mean spectral power differences (CS+ minus CS-) in the time-frequency domain across the cluster sensors for each experimental phase are shown. The colorbar represents power changes in dB.

Both, the bandpass and only highpass filtered C1 responses were not modulated by conditions and experimental phase (see Figure 2S B, , biggest summed F= 541; p = 0.173 for the bandpass filtered C1 and summed F = 94, p = 0.737 for the highpass filtered C1). Similarly, in the lower frequency bands (6 Hz to 40 Hz) no interaction of conditioning and experimental phase was observed below 18 Hz (biggest summed F = 4550, p = 0.107; Figure 2S C). In Figure 2S we show the mean C1 amplitude across the significant sensor cluster for the evoked gamma band effects and mean power changes in the lower frequency bands to illustrate that the evoked gamma band modulations at these sensor sites were not driven by C1 and lower frequency bands.


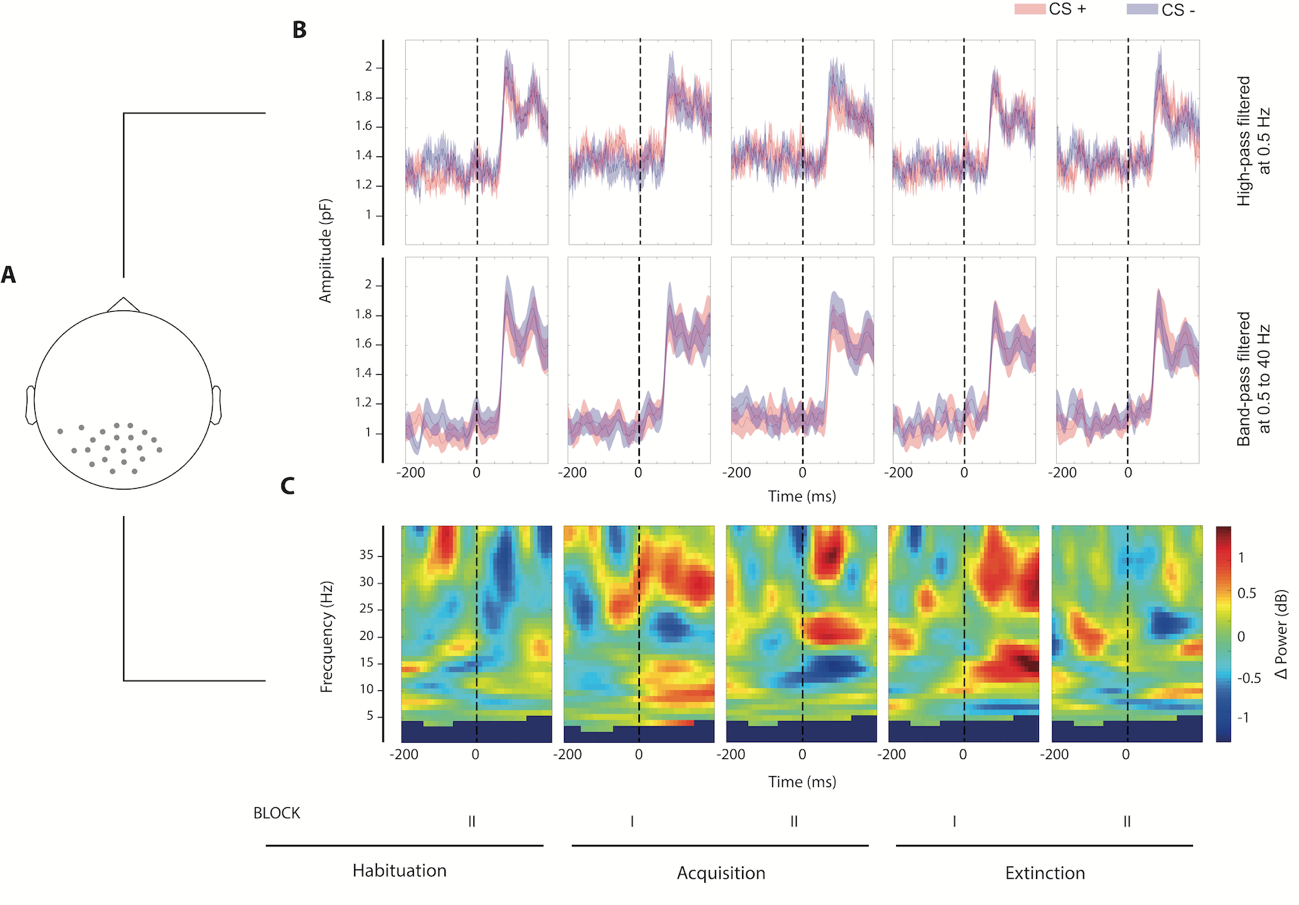


**Figure 2S:** Mean C1 amplitude and power changes in the lower frequency bands (6 Hz to 40 Hz) are shown across the previously reported significant sensor cluster to illustrate that evoked gamma band power modulations were not driven by C1 amplitude and lower frequency components. **(A)** The sensor cluster of significant evoked gamma power modulation (see Figure 1, main text) is shown. **(B)** The upper panel shows the time domain magnetic counterpart of the evoked C1 response (highpass filter 0.5 Hz). The lower panel shows the same for the bandpass filtered (0.5 Hz to 40 Hz) evoked field. The shaded areas represent the standard errors. **(C)** Time-frequency spectral perturbations are shown for the lower frequency bands (6 Hz to 40 Hz). Note, that the upper limits (from 15 Hz on) overlap with the original analysis (see main text).

Although, during the habituation phase, the CS- evoked greater evoked gamma power changes, CS+ related gamma power was still greater than the CS- gamma band response during the second acquisition phase (see main text for statistics). Figure 3S depicts baseline corrected (dB) gamma power changes for the CS+ and CS- condition during the second acquisition phase.


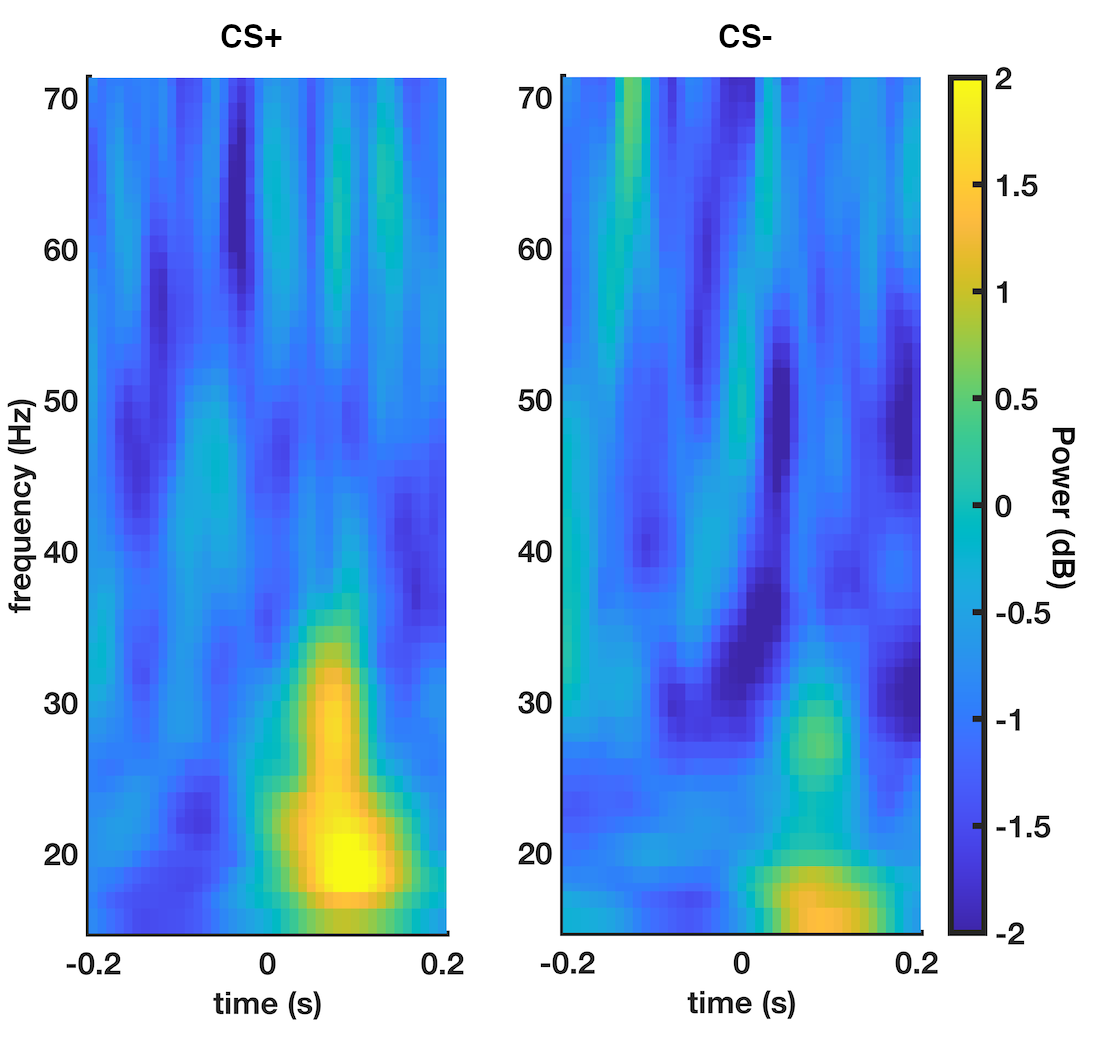


**Figure 3S:** Visual evoked gamma band response (dB with respect to baseline) for the CS+ and CS- condition during the second acquisition phase are shown, where the learning effect was greatest (see Figure 1 main text).

In order to asses a neurophysiological index of attention, the alpha band power modulation for the CS+ and CS- in each experimental phase was also evaluated. No interaction of experimental phase (habituation II, acquisition I & II, extinction I & II) and condition (CS+, CS-) with respect to induced alpha power changes (dB) (F(4, 116) = 0.255, p = 0.856, ε = 0.79, η^2^ = 0.009) was observed (see Figure 4S).


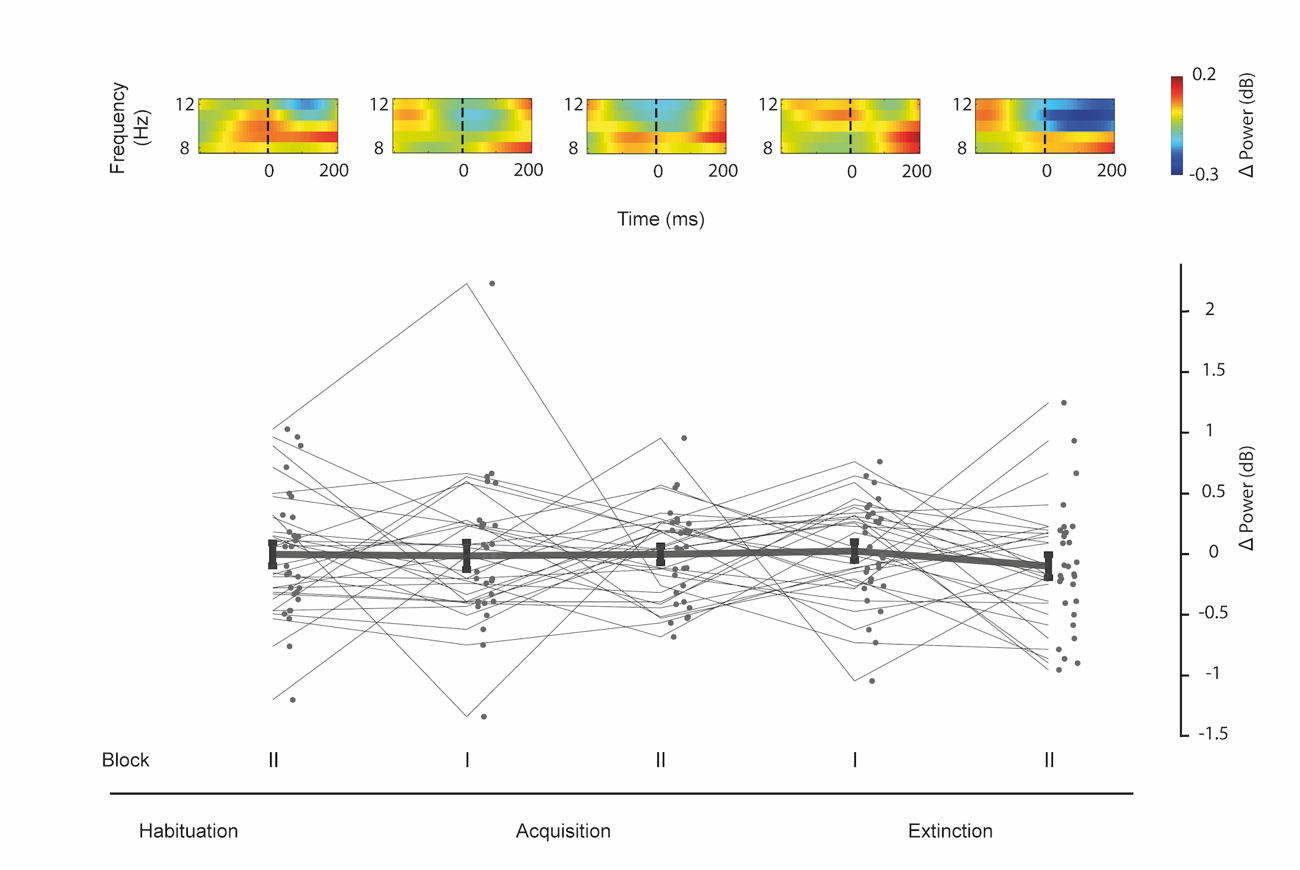


**Figure 4S:** Induced power changes (dB) in the alpha band frequency range (8 Hz to 12 Hz) are shown in the upper row. Below, the paired observation plot for the mean alpha power changes across all frequency bins (8 Hz to 12 Hz), post-stimulus interval time bins (0 ms to 200 ms) and the posterior sensor cluster is shown. The thick grey line indicates the mean difference between the CS+ and the CS- across subjects. The error bars represent the standard error of this difference. The connected grey dots represent single subject differences between the CS+ and the CS-.
